# Sleep Loss Produces False Memories

**DOI:** 10.1371/journal.pone.0003512

**Published:** 2008-10-23

**Authors:** Susanne Diekelmann, Hans-Peter Landolt, Olaf Lahl, Jan Born, Ullrich Wagner

**Affiliations:** 1 Department of Neuroendocrinology, University of Lübeck, Lübeck, Germany; 2 Institute of Pharmacology and Toxicology, and Zürich Center for Integrative Human Physiology (ZIHP), University of Zürich, Zürich, Switzerland; 3 Institute of Experimental Psychology, Heinrich-Heine-University Düsseldorf, Düsseldorf, Germany; 4 Department of Neuroscience, University Medical School, University of Geneva, Geneva, Switzerland; Victoria University of Wellington, New Zealand

## Abstract

People sometimes claim with high confidence to remember events that in fact never happened, typically due to strong semantic associations with actually encoded events. Sleep is known to provide optimal neurobiological conditions for consolidation of memories for long-term storage, whereas sleep deprivation acutely impairs retrieval of stored memories. Here, focusing on the role of sleep-related memory processes, we tested whether false memories can be created (a) as enduring memory representations due to a consolidation-associated reorganization of new memory representations during post-learning sleep and/or (b) as an acute retrieval-related phenomenon induced by sleep deprivation at memory testing. According to the Deese, Roediger, McDermott (DRM) false memory paradigm, subjects learned lists of semantically associated words (e.g., “night”, “dark”, “coal”,…), lacking the strongest common associate or theme word (here: “black”). Subjects either slept or stayed awake immediately after learning, and they were either sleep deprived or not at recognition testing 9, 33, or 44 hours after learning. Sleep deprivation at retrieval, but not sleep following learning, critically enhanced false memories of theme words. This effect was abolished by caffeine administration prior to retrieval, indicating that adenosinergic mechanisms can contribute to the generation of false memories associated with sleep loss.

## Introduction

A fundamental finding of memory research has been that human memory is not a literal record of the world, but is influenced by knowledge representations that already exist in the brain [1]. Consequently, what is retrieved from memory can substantially differ from what was originally encoded [2], [3]. One particularly interesting example are false memories, i.e. when people claim to remember events that in fact never happened. Typically, false memories are semantically strongly associated to actually encoded events, and subjects are highly confident about the correctness of these memories [4]–[7]. It can be assumed that the development of false memories follows the same basic principles of memory formation as the development of correct memories, comprising the three different sub-processes encoding (learning), consolidation (off-line processing and strengthening of memory traces after encoding), and retrieval of the learned material [3].

Sleep represents a neurobiological condition that is critically involved in memory formation [8]. Specifically, sleep plays an active role in memory consolidation [9]–[12]. During sleep, newly acquired memory traces are not only strengthened in distinct neural circuits (synaptic consolidation), but fresh memory traces are also redistributed to other brain regions for long-term storage and integrated within pre-existing long-term memories, a process termed system consolidation [13]–[15]. This active restructuring may also lead to the formation of false memories, because after active reorganization and integration within pre-existing representations, the memory representation can qualitatively differ from what was originally encoded [16], [17]. In this case, false memories would be created during consolidation as new and enduring knowledge representations, which are ‘false’ in the sense that they abstract from the actually encoded material by generalizing to semantically associated knowledge. By this way, sleep itself may promote false memories during memory consolidation.

On the other hand, the occurrence of false memories could result from acute disturbances in the retrieval process that do not rely on ‘false’ representations per se. In this case, prolonged *loss* of sleep would be expected to enhance false memories. Ample evidence indicates that sleep deprivation markedly impairs cognitive functions like vigilance, attention, working memory, divergent thinking and other executive functions [18], [19]. Importantly, memory retrieval is likewise acutely impaired under sleep deprivation, which has been attributed to reduced source and reality monitoring [18], [20]–[22], and the same mechanisms may also acutely support the generation of false memories.

A series of four experiments was performed to test these hypotheses, applying the well-established Deese, Roediger, McDermott (DRM) false memory paradigm [4], [23], which uses word lists reliably yielding unusually high amounts of false memories [6], [7], [24]. Subjects learned lists of semantically associated words (e.g. “night”, “dark”, “coal”,…). The strongest associate, however, the “theme” of the list - “black” in this example - was not presented during learning. At retrieval testing, 9, 33 or 44 hours after learning, list words were presented again together with the “theme” word (or “critical lure”) and unrelated distractor words, and subjects had to indicate for each word whether it was presented during learning or not (recognition test). Subjects either slept or stayed awake in the consolidation phase immediately following learning, and they were or were not acutely sleep deprived at retrieval. Sleep deprivation at retrieval, but not sleep after learning, substantially enhanced the proportion of false memories. This effect was neutralized when caffeine was administered before retrieval testing, indicating that adenosinergic mechanisms can contribute to false memory generation following sleep loss.

## Results

### Memory performance

#### Experiment I

**Figure 1:**
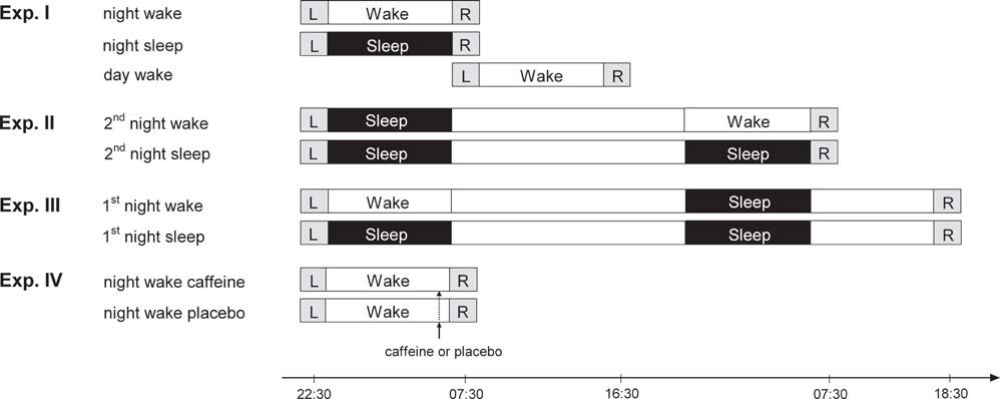
Experimental design. Subjects either slept or stayed awake in the consolidation phase following learning, and either were or were not sleep deprived at retrieval. Black fields refer to sleep periods; blank fields represent times of wakefulness. Times of learning (L) and retrieval (R) are indicated for Experiments I to IV.

We first compared false memory rates in three groups of subjects with a delay of 9 hours between learning and retrieval testing (Figure 1). Two groups learned in the evening and were tested the next morning, after they had slept (“night sleep”, n = 15) or stayed awake during the intervening night (“night wake”, n = 14). The third group (“day wake”, n = 14) learned in the morning and was tested in the same evening after normal daytime wakefulness.

**Figure 2:**
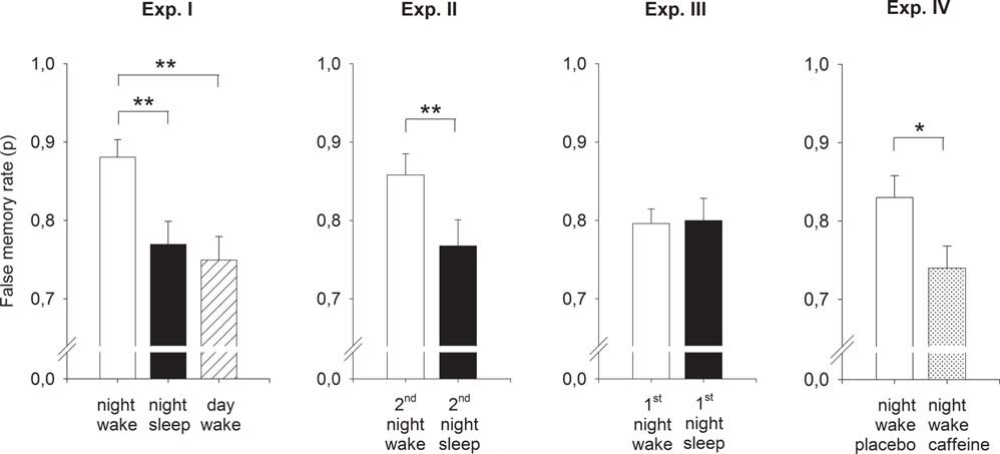
Proportion of false memories in the recognition test. Under sleep deprivation at retrieval false memory rate was significantly enhanced in Experiment I (higher false memory rate in the sleep deprived “night wake” group compared to both non-deprived groups), while sleep after learning compared to wakefulness did not increase false memories (no difference between the “night sleep” and “day wake” group). Experiments II and III further strengthen these findings in showing that sleep deprivation at retrieval also enhanced false memory rate when “sleep vs. wakefulness after learning” was held constant and subjects only were or were not sleep deprived at retrieval (“2^nd^ night wake” vs. “2^nd^ night sleep” in Experiment II), and that sleep after learning neither enhanced false memories when retrieval was tested after a recovery night and controlling for circadian phase (“1^st^ night wake” vs. “1^st^ night sleep” in Experiment III). The administration of caffeine one hour before retrieval testing in Experiment IV abolished the sleep deprivation-induced enhancement in false memories. False memory rate refers to the mean proportion of the judgment “old” to 18 theme words that were not presented during learning (mean±SEM). * P<0.05, ** P<0.01.

Subjects of the “night wake” group who were acutely sleep deprived at retrieval testing exhibited significantly more false memories than subjects in the two other groups [F (2, 40) = 6.90; P = 0.003; Table 1; Figure 2]. After nocturnal wakefulness the proportion of falsely recognized theme words was on average 0.88±0.02, i.e. subjects falsely recognized 88% of the theme words, whereas after sleep and diurnal wakefulness false memory rate was 0.77±0.03 and 0.75±0.03, respectively (mean±SEM) [t (27) = 3.40, P = 0.002 and t (27) = 4.01, P<0.001, for pair-wise comparisons]. Importantly, subjects did not produce more false memories in the “night sleep” group than in the “day wake” group, which would be expected if consolidation processes during post-learning sleep were critical for the development of false memories [t (26) = 0.46, P>0.60]. There was no difference between the three groups in hit rates (correctly recognized words) [F (2, 40) = 0.78, P>0.40] and false alarm rates (falsely recognized distractor words) [F (2, 40) = 1.18, P>0.30] (Table 1). To exclude that increased false memory generation after sleep deprivation merely resulted from enhanced baseline propensity to accept items, additional analyses were performed with the discrimination index P*_r_* and the response bias index B*_r_* according to the two-high threshold model of recognition memory as dependent variables ([25]; see Materials and Methods, for details). In these analyses, sleep deprived subjects likewise exhibited significantly more false memories compared to both non-deprived control groups [P*_r_* = 0.67±0.04, 0.50±0.04 and 0.47±0.04, for the “night wake”, “night sleep” and “day wake” group, respectively; F (2, 40) = 8.69, P = 0.001], whereas correct recognition memory again did not differ between groups [P*_r_* = 0.51±0.04, 0.40±0.04 and 0.42±0.04, for the “night wake”, “night sleep” and “day wake” group, respectively; F (2, 40) = 2.05, P>0.14]. The response bias, either for false recognition or correct recognition, also did not differ between groups [false recognition: B*_r_* = 0.63±0.06, 0.55±0.06 and 0.53±0.06; correct recognition: B*_r_* = 0.40±0.04, 0.45±0.04 and 0.47±0.04; both F (2, 40)<1.20, P>0.30].

**Table 1:** Recognition memory performance, confidence ratings and remember/know/guess judgments.

	Exp. I			Exp. II		Exp. III		Exp. IV	
	night wake	night sleep	day wake	2^nd^ night wake	2^nd^ night sleep	1^st^ night wake	1^st^ night sleep	night wake placebo	night wake caffeine
Recognition									
False memories	0.88±0.02**	0.77±0.03	0.75±0.03	0.86±0.02*	0.76±0.03	0.80±0.03	0.80±0.02	0.83±0.03*	0.74±0.03
Hits	0.72±0.03	0.67±0.02	0.70±0.03	0.68±0.02	0.70±0.02	0.66±0.03	0.61±0.03	0.73±0.02	0.66±0.04
False alarms	0.21±0.04	0.27±0.03	0.28±0.03	0.33±0.03	0.29±0.03	0.35±0.04	0.33±0.04	0.28±0.04	0.27±0.04
Confidence									
False memories	3.54±0.08	3.33±0.11	3.26±0.10	3.27±0.11	3.32±0.09	3.24±0.09	3.24±0.09	3.30±0.09	3.25±0.10
Hits	3.39±0.09	3.22±0.12	3.21±0.08	3.19±0.08	3.19±0.08	3.14±0.06	3.18±0.06	3.29±0.07	3.31±0.07
False alarms	2.70±0.15	2.45±0.14	2.28±0.12	2.65±0.13	2.61±0.08	2.49±0.12	2.37±0.12	2.41±0.12	2.47±0.14
Remember									
False memories	0.41±0.07	0.38±0.07	0.37±0.06	0.45±0.07	0.43±0.05	0.43±0.04	0.39±0.05	0.40±0.06	0.38±0.06
Hits	0.45±0.06	0.38±0.06	0.42±0.04	0.43±0.04	0.39±0.04	0.39±0.04	0.35±0.05	0.45±0.04	0.42±0.05
False alarms	0.11±0.04	0.06±0.03	0.08±0.03	0.25±0.04	0.18±0.03	0.19±0.04	0.14±0.04	0.13±0.05	0.18±0.06
Know									
False memories	0.39±0.05	0.38±0.06	0.35±0.03	0.33±0.06	0.36±0.05	0.35±0.04	0.38±0.05	0.34±0.05	0.34±0.05
Hits	0.30±0.04	0.29±0.05	0.29±0.02	0.31±0.05	0.37±0.04	0.35±0.04	0.40±0.04	0.28±0.03	0.32±0.03
False alarms	0.29±0.05	0.19±0.05	0.26±0.07	0.26±0.04	0.39±0.06	0.32±0.05	0.23±0.04	0.27±0.03	0.29±0.07
Guess									
False memories	0.20±0.04	0.29±0.05	0.28±0.05	0.22±0.04	0.21±0.04	0.24±0.05	0.23±0.05	0.26±0.05	0.27±0.05
Hits	0.25±0.03	0.33±0.04	0.30±0.04	0.23±0.03	0.24±0.03	0.25±0.03	0.26±0.02	0.27±0.03	0.26±0.04
False alarms	0.61±0.08	0.75±0.06	0.67±0.07	0.46±0.05	0.43±0.06	0.50±0.06	0.63±0.06	0.59±0.08	0.53±0.09

Recognition is indicated by the mean proportion of “old” judgments on theme words ( = False memories), list words ( = Hits) and distractors ( = False alarms). Mean confidence ratings (ranging from 1 = “guess” to 4 = “sure”) and proportions of Remember, Know and Guess judgments are displayed for words judged as “old”. Means±SEM are shown. ^*^ P<0.05, ^**^ P<0.01, compared to respective control groups within each experiment.

This pattern of results indicates that sleep deprivation at retrieval, but not sleep after learning, critically enhances false memories. Experiments II and III were designed to further strengthen this conclusion by separating the two factors “sleep-deprived vs. non-deprived state at retrieval” and “sleep vs. wakefulness after learning”, and carefully controlling for possible circadian influences.

#### Experiment II

Only the factor “sleep-deprived vs. non-deprived state at retrieval” was manipulated, while the factor “sleep vs. wakefulness after learning” was held constant. Two groups of subjects learned in the evening and slept in the first night after learning. In the second night after learning, one group stayed awake to be sleep-deprived at retrieval testing on the next morning (“2^nd^ night wake”, n = 15), whereas the other group slept normally (“2^nd^ night sleep”, n = 16; Figure 1). In view of the results from Experiment I, we expected enhanced false memory retrieval under sleep deprivation, compared to the non-deprived state, also when retrieval testing took place one day later.

In fact, subjects who were sleep deprived at retrieval (“2^nd^ night wake”), like in Experiment I, showed higher false memory rates than non-sleep deprived subjects [0.86±0.02 vs. 0.76±0.03; t (35) = 2.62, P = 0.007; Figure 2]. As in Experiment I, hit rate [t (35) = −0.85, P>0.40] and false alarm rate [t (35) = 1.05, P = 0.30] did not differ between the groups (Table 1). False recognition corrected for baseline propensity to accept items was likewise higher in sleep deprived than in non-deprived subjects [P*_r_* = 0.53±0.04 vs. 0.48±0.05, for the “2^nd^ night wake” and “2^nd^ night sleep” group, respectively; t (35) = 1.68, P = 0.05]. Like in Experiment I, correct recognition scores [P*_r_* = 0.35±0.03 and 0.42±0.03, t (35) = −1.39, p>0.17], as well as bias indices for both false recognition and correct recognition did not differ between groups [false recognition: B*_r_* = 0.72±0.04 and 0.54±0.05; correct recognition: B*_r_* = 0.50±0.03 and 0.46±0.04; both t (35)<1.20, P>0.20].

#### Experiment III

Only the factor “sleep vs. wakefulness after learning” was manipulated, while all subjects were not sleep-deprived at retrieval. Two groups of subjects learned in the evening, and in the subsequent night either slept normally (“1^st^ night sleep”, n = 15) or stayed awake (“1^st^ night wake”, n = 17). All subjects slept in the second night after learning and were tested for retrieval in the evening thereafter (Figure 1). In this way, not only were sleep/wake times and testing times paralleled between experimental groups, but times of learning and retrieval were also the same *within* subjects.

Confirming the results of Experiment I, false memory rate did not differ between subjects in the “1^st^ night sleep” and the “1^st^ night wake” groups [0.80±0.02 vs. 0.80±0.03; t (30) = −0.12, P>0.90; Figure 2]. Again, the groups did not differ in hit rate [t (30) = −1.13, P>0.20] and false alarm rate [t (30) = −0.51, P>0.60] (Table 1), as well as in baseline-corrected false recognition [P*_r_* = 0.47±0.04 and 0.44±0.04, for the “1^st^ night sleep” and “1^st^ night wake” group, respectively; t (30) = 0.29, p>0.70] and correct recognition [P*_r_* = 0.28±0.03 and 0.30±0.04, t (30) = −0.16, p>0.80]. Bias indices were also comparable between groups [false recognition: B*_r_* = 0.60±0.04 and 0.66±0.05; correct recognition: B*_r_* = 0.45±0.05 and 0.51±0.04; both t (30)<−0.50, P>0.60].

#### Experiment IV

In view of the results from Experiments I to III, which consistently show that it is specifically sleep deprivation at retrieval that renders subjects susceptible to false memories, Experiment IV was performed to explore a possible neurophysiological mechanism underlying this effect. Adenosinergic activity is thought to play a key role in the emergence of sleepiness and impairment of executive cognitive functions after prolonged wakefulness [26]–[28]. Based on this background we hypothesized that caffeine, an adenosine receptor antagonist, reduces the occurrence of false memories after sleep deprivation. Two groups of subjects learned in the evening, stayed awake during the night and were tested again the next morning as in the “night wake” group of Experiment I (Figure 1). One group received 200 mg caffeine one hour before the start of retrieval testing (“night wake caffeine”, n = 15) and the other group received placebo (“night wake placebo”, n = 18). Caffeine and placebo were administered according to a randomized, double-blind design.

The “night wake caffeine” group exhibited a significantly lower false memory rate when compared to the “night wake placebo” group [0.74±0.03 vs. 0.83±0.03; t (31) = −2.41, P = 0.022; Figure 2]. Again, in contrast to false memory rate, hit rate [t (31) = −1.47, P>0.15] and false alarm rate [t (31) = −0.20, P>0.80] did not differ between groups (Table 1), and again the same pattern occurred when baseline-corrected measures were used: False recognition was significantly reduced after caffeine administration [P*_r_* = 0.47±0.04 vs. 0.55±0.03, for the “night wake caffeine” and “night wake placebo” group, respectively, t (31) = −2.43, P = 0.021], whereas scores of correct recognition did not differ between groups [P*_r_* = 0.39±0.03 vs. 0.45±0.04, t (31) = −0.76, P>0.40]. The response bias per se, like in the previous experiments, did not differ between groups, neither for false recognition nor for correct recognition [false recognition: B*_r_* = 0.49±0.05 and 0.61±0.06; correct recognition: B*_r_* = 0.44±0.06 and 0.48±0.05; both t (31)<−0.20, P>0.70].

### Confidence ratings and remember/know/guess judgments in recognition memory

All subjects tested in Experiments I to IV gave confidence ratings for their answers in the recognition memory test, as well as remember/know/guess judgments for the positive answers [4], [29]. Confidence ratings were in all experiments distinctly higher for theme words and list words when compared to distractors (main effects “word type”, all P<0.001; pair-wise comparisons, all P<0.001). Also the proportion of “remember” judgments was higher for theme words and list words than for distractors in all experiments (main effects “word type”, all P<0.001; pair-wise comparisons, all P<0.001). However, in none of the experiments differed the groups significantly in these variables (main effects “group” and interactions “group×word type”, all P>0.10; Table 1), indicating that sleep deprived subjects did not exhibit higher confidence or more remember judgments on false memories, hits or false alarms compared to non-deprived controls.

### Control variables

#### Subjective ratings

Subjects in all experiments rated their subjective sleepiness, activation, motivation and concentration immediately before learning and recognition testing. As expected, sleep-deprived groups (i.e. the “night wake” group in Experiment I and the “2^nd^ night wake” group in Experiment II) scored higher at retrieval in subjective ratings of sleepiness, and lower in motivation and concentration than the respective non-sleep deprived groups (all P<0.05; Table 2). In Experiment I, at learning subjects in the “night sleep” group were also sleepier and less activated than the “night wake” and “day wake” group (P<0.05), possibly because subjects anticipated they would be allowed to sleep soon. In Experiment IV, caffeine administration after sleep deprivation significantly reduced subjective sleepiness and increased feelings of activation and motivation compared to placebo (P≤0.05; Table 2).

**Table 2:** Subjective ratings at learning and retrieval.

	Exp. I			Exp. II		Exp. III		Exp. IV	
	night wake	night sleep	day wake	2^nd^ night wake	2^nd^ night sleep	1^st^night wake	1^st^ night sleep	night wake placebo	night wake caffeine
Learning									
Sleepiness	2.53±0.32	3.36±0.25*	2.00±0.26	2.11±0.21	2.33±0.21	2.47±0.23	3.20±0.30	2.17±0.20	2.13±0.27
Activation	3.33±0.19	2.57±0.29*	3.36±0.23	3.39±0.20	3.50±0.23	3.00±0.21	2.60±0.13	3.67±0.20	3.40±0.19
Motivation	3.40±0.19	3.36±0.20	3.57±0.20	3.61±0.22	3.94±0.13	3.06±0.18	3.07±0.23	3.67±0.18	3.47±0.17
Concentration	3.00±0.17	2.93±0.22	3.57±0.23	3.50±0.20	3.22±0.15	3.00±0.17	2.93±0.15	3.33±0.23	3.67±0.23
Retrieval									
Sleepiness	3.67±0.30**	2.36±0.27	1.93±0.29	4.17±0.25**	2.26±0.25	1.94±0.29	1.80±0.20	4.50±0.26	3.20±0.35**
Activation	2.07±0.18**	3.14±0.21	3.57±0.29	1.78±0.15**	3.53±0.21	3.23±0.25	3.53±0.17	2.22±0.22	3.07±0.32*
Motivation	2.80±0.17*	3.64±0.17	3.50±0.29	2.00±0.20**	3.47±0.16	3.35±0.21	3.53±0.22	2.33±0.24	3.07±0.27*
Concentration	2.27±0.12**	3.29±0.13	3.21±0.24	2.22±0.21**	3.37±0.14	3.35±0.26	3.53±0.24	2.33±0.18	2.73±0.25

Subjective ratings ranged from 1 = “not at all” to 5 = “very much”. ^*^ P≤0.05, ^**^ P<0.01, compared to respective control groups within each experiment.

#### Sleep data

For experimental conditions involving sleep, quality of sleep was controlled by standard polysomnography (Experiments I and III) and sleep questionnaires (Experiments II and III). In Experiment I, polysomnographic recordings of the “night sleep” group revealed normal sleep patterns with a total sleep time of 411.3±4.4 min (mean±SEM; sleep stage 1, 4.4±0.80%; sleep stage 2, 55.8±1.7%; slow wave sleep, 18.5±1.7% and rapid eye movement sleep, 20.8±1.5%). In Experiment II, subjects slept at home during the first night after learning and according to sleep questionnaire data, subjects in the “2^nd^ night sleep” and “2^nd^ night wake” group did not differ significantly in the time they went to bed (23:57 h±18 min vs. 00:39 h±13 min) or total sleep time (7.64±0.32 h vs. 7.86±0.25 h) (P>0.05). In the second night, subjects in the “2^nd^ night sleep” condition went to bed on average at 00:42 h±17 min and slept for 6.54±0.28 h. In Experiment III, subjects of the “1^st^ night sleep” group slept in the sleep laboratory during the first night after learning and recordings revealed normal sleep (total sleep time, 441.7±7.7 min; sleep stage 1, 4.3±0.54%; sleep stage 2, 49.1±2.9%; slow wave sleep, 21.3±3.0%, rapid eye-movement sleep, 23.8±2.0%). In the second night (recovery night for subjects in the “1^st^ night wake” group) all subjects slept at home and filled in sleep questionnaires. Subjects in the “1^st^ night wake” group went to bed significantly earlier than the “1^st^ night sleep” group (22:53±32 min vs. 00:04±44 min, P<0.05). The groups did not differ significantly in mean sleep time (9.97±0.50 h vs. 8.95±0.38 h for “1^st^ night wake” and “1^st^ night sleep” groups, respectively; P>0.10).

#### Salivary cortisol

Salivary cortisol concentrations were measured at retrieval in Experiment I on the background of previous studies indicating substantial influences of corticosteroids on memory retrieval (e.g., [30]) and of psychosocial stress on false recognition ([31], but see [32]). Cortisol concentrations (in nmol/l and collapsed across measurements before, during and after retrieval) differed significantly between the groups [F (2, 40) = 23.97, P<0.01] with a mean of 23.66±2.88 in the “night sleep” group, 11.12±2.06 in the “night wake” group, and 5.45±0.77 in the “day wake” group. The differences reflect the typical circadian variation in salivary cortisol levels and the cortisol response after awakening in the night sleep group [33], [34]. Individual cortisol concentrations did not correlate with false memory rate, hit rate or false alarm rate (r<0.14, P>0.30). Thus, sleep deprivation is unlikely to have affected false memories by stress-associated alterations.

## Discussion

We investigated sleep-associated mechanisms of false memory generation in the DRM false memory paradigm. A series of experiments was performed to test whether consolidation sleep following learning increases false memories, and/or whether acute sleep deprivation at retrieval testing does so. Results from Experiments I to III provide strong evidence for the latter rather than the former hypothesis. Sleep deprivation at retrieval testing, but not sleep after learning, critically enhanced the rate of false memories. In addition, Experiment IV showed that this effect can be neutralized by administration of caffeine before retrieval testing.

It could be argued that false memory rates were higher in sleep deprived subjects simply due to loss of motivation or reduced compliance. If this were true, however, hit rate and false alarm rate should have been similarly affected. This was not the case. Moreover, lower confidence ratings and more judgments of guessing would be expected with reduced motivation to engage in the task. Also in these variables, the sleep deprived subjects did not differ from the non-sleep deprived subjects. Sleep deprivation likewise did not change the response criterion subjects adopted to make old-new decisions in the recognition task, and sleep deprivation also enhanced false memories when corrected for response bias, indicating that higher false memory rates in sleep deprived subjects are not attributable simply to a more liberal response criterion (although a minor contribution of this factor cannot be entirely ruled out). Thus, we conclude that the observed differences primarily derive from changes in brain functions genuinely linked to the sleep deprived state. This conclusion is further supported by the finding that blocking adenosine receptors by caffeine at retrieval counteracts false memory enhancement in sleep-deprived subjects.

Ample evidence indicates that sleep deprivation strongly affects cognitive functions essentially relying on the integrity of the prefrontal cortex (PFC) [18]–[21], [35]. Notably, the PFC has been specifically implicated in false recognition [36]–[38]. False memories, as compared to true memories, show greater activation in prefrontal regions, especially in the right PFC [24]. These regions have been associated with effortful aspects of retrieval involving inhibition, post-retrieval monitoring, criterion setting and decision making about the sense of familiarity or recollection associated with false recognition [24], [39]–[41]. One study suggested that PFC activation is required to limit or avoid false recognition [42]. In this study, Curran and colleagues compared event-related potentials (ERP) in subjects who discriminated well between studied and non-studied items (good performers) and subjects who did not (poor performers). Good performers were characterized by a more positive late right frontal ERP than poor performers. This finding possibly reflects retrieval monitoring processes that are more likely engaged in good performers than in poor performers. The impairment of prefrontal lobe function after sleep deprivation may derogate these kinds of reality monitoring, which are necessary to determine whether or not a word was actually encountered before or internally generated [43], [44]. Assuming that sleep deprivation specifically impairs the ability to discriminate previously encountered words from new words, it could be expected that not only false memories, but also false alarms on distractors that are not semantically associated should be enhanced following sleep deprivation. This was not the case here. However, discriminating presented list words from highly associated theme words is much more difficult than distinguishing old list words from non-associated distractors and thus is possibly more prone to cognitive impairments associated with sleep loss.

Regarding the underlying neurophysiological mechanisms, our caffeine experiment points to adenosine as one factor involved in the decline of these cognitive functions under sleep deprivation. This notion is in line with previous data pointing to a key role for adenosinergic neuromodulation in the emergence of sleepiness and impairment of neurobehavioral functions after prolonged wakefulness [26]–[28]. Caffeine acts as an antagonist at adenosine receptors [45]. By blocking adenosine A_1_ receptor-mediated neuronal inhibition, caffeine increases cortical and hippocampal activity [46], and studies in rats showed that it induces acetylcholine release in prefrontal areas [47]. Such mechanisms may underlie the enhanced effectiveness in prefrontal functioning after caffeine administration and might in this way have mediated the caffeine-induced reduction of false memories in sleep-deprived subjects.

It has to be noted, however, that changes in prefrontal functioning are not the only possible explanation for the occurrence of false memories after sleep loss. Indeed, other brain regions and cognitive functions related to memory are likewise negatively affected by sleep loss [19], [48]. Specifically, sustained attention and arousal, relying on a prefrontal-parietal network as well as on the basal forebrain and thalamus, are substantially reduced under sleep deprivation and are known to be implicated in memory functions [49]–[52]. It is presently not clear how arousal and attention interact specifically in relation to false memory generation. False memories have recently been shown to be enhanced when attentional resources at retrieval are reduced [53]. On the other hand, false memory rates are lower with reduced arousal and enhanced in high arousal conditions, e.g. following psychosocial stress [31] or emotional arousal [54]. Caffeine increases arousal and enhances attentional resources [55], as confirmed also by the subjective data here, which could at least partly account for the reduced occurrence of false memories with caffeine. Because we did not test the specificity of the observed effect for caffeine, it remains to be elucidated whether acting on arousal and attention by other stimulants not specifically targeting the adenosinergic system (e.g., modafinil) can similarly reduce false memories.

In contrast to the acute retrieval-related effects induced by sleep deprivation, our findings do not point to a critical contribution of system consolidation during post-learning sleep to the generation of false memories. Sleep after learning did not increase false memories compared to post-learning wakefulness even when cognitive state at retrieval and circadian influences were controlled for (Experiment III). It has to be noted, however, that not only false memory rate, but also hit rate (true recognition) was unaffected by post-learning sleep when compared to wakefulness. One possible reason is the kind of memory testing used here, i.e. recognition memory. Although consolidation effects of sleep following learning were previously found within recognition memory tasks [56] some studies suggest that recognition memory appears to be less affected by post-learning sleep than free recall or cued recall [57], [58]. Thus, it is conceivable that effects of post-learning sleep on the generation of false memories could be revealed with more sensitive testing procedures. Future studies should address this issue directly by using procedures of free recall or cued recall in the same experimental paradigm as used here.

In sum, we found that acute sleep deprivation increases false memories, while sleep after learning did not influence false memory formation. Although false memories are formally a kind of memory distortion, for proper adaptation it might be useful especially in situations of restricted cognitive control (as in a state of sleep deprivation) to rely on the gist of a memory, i.e. the broader semantical network associated with actually experienced events. In other cases, however, an exact distinction between closely related memory representations is crucial, e.g. in eyewitness testimony. Apart from other factors that can produce distortions of memory retrieval (e.g., suggestive interview procedures; see [59], for an overview), our results clearly show that sleep deprivation is another critical factor that must be avoided in such situations.

## Materials and Methods

### Participants

A total of 145 healthy adults [age 23.7±3.2 (mean±SD), range 18–35 yr, 59 females] with regular sleep-wake cycles (≥6 hours sleep per night) and no shift work for at least six weeks prior to the experiments participated in the study. Subjects were not allowed to ingest any caffeine or alcohol from the day before until the end of the experiments. Prior to experimental nights all subjects spent an adaptation night in the sleep laboratory. Subjects in Experiment IV were moderate caffeine consumers (<250 mg per day). They had to rate themselves as being caffeine-sensitive, and to abstain from caffeine for two weeks prior to the experiment to exclude withdrawal effects. All subjects gave written informed consent and were paid for participation in the study, which was approved by the local ethics committee of the University of Lübeck.

### False memory task

The standard Deese, Roediger & McDermott (DRM) procedure [4], [23] was used to induce false memories. All subjects learned 18 DRM lists, selected from Stadler et al. [60] and translated into German. Each list consisted of 15 semantically associated words (e.g. “night”, “dark”, “coal”,…). The strongest associate, however, i.e. the “theme” of the list (“black” in this example) was not presented during learning. For each list, words were presented in the order from the strongest to the weakest associative strength with respect to the theme word. The list words were recorded electronically in a female voice and presented once sequentially with a delay of ten seconds between lists and 750 ms between words. Subjects learned individually in a sound-attenuated room where the words were presented via loudspeakers. They were instructed to pay attention to the words and to memorize them as accurately as possible because memory would be tested later.

At retrieval testing, recognition memory was tested by a computerized programme. Three types of words were presented: list words (actually presented during learning, specifically the words of serial position 1, 5 and 10 of each list), unrelated distractors (which were not presented during learning and not associated to the list words; specifically, these words were list words from other DRM lists, e.g. “highway” and “tall” for the theme word “black”) and, as “critical lures”, the theme words of the lists (that had not been presented during learning, but were semantically strongly associated to the list words). Words were presented visually in white letters on a black background in the middle of a 17″ computer screen. Altogether, 108 words (54 list words, 36 distractors and 18 theme words) were presented to the subjects. For each word they had to give an old/new judgment (i.e. to indicate whether the word had been presented during learning or not) and a confidence rating for their answer on a 4-point scale ranging from 1 (“I had to guess”) to 4 (“absolutely sure”) by clicking with the mouse on the corresponding buttons. After all 108 words were presented, those words that were previously judged as “old” were presented again, and the participants gave a Remember/Know/Guess (RKG) judgment according to established procedures [7], [29]. There was no time limit for any judgments.

### Design and procedure

A series of four experiments was performed to disentangle factors related to consolidation and retrieval in sleep-associated false memory generation, carefully controlling for possible circadian influences on memory formation [61] (Exp. I to III) and exploring possible underlying neurophysiological mechanisms in a pharmacological study (Exp. IV). An overview of the experimental designs used in these experiments is given in Figure 1.

#### Experiment I

This initial experiment compared false memory rates in three groups of subjects with a delay of 9 hours between learning and retrieval testing, where the effect of regular sleep in the consolidation phase following learning (“night sleep”) was compared with two wake conditions, one with (“night wake”) and one without (“day wake”) acute sleep deprivation at retrieval testing. In the “night sleep” and “night wake” group subjects learned at 22:30 h. Thereafter, subjects in the “night sleep” group were allowed to sleep from 23:00 to 07:00 h in the sleep laboratory with standard polysomnographic recordings. Subjects in the “night wake” condition stayed awake in the laboratory and were allowed to read, watch TV and play simple games. Retrieval testing in both groups was performed at 07:30 h. For the “day wake” group learning and retrieval occurred at 08:00 h and 17:00 h. During the retention interval they engaged in everyday activities, which they reported in a questionnaire afterwards.

#### Experiment II

By comparing a “2^nd^ night wake” and a “2^nd^ night sleep” group, with normal sleep in the night following learning for all subjects, only the factor “sleep-deprived vs. non-deprived state at retrieval” was manipulated, while the factor “sleep vs. wakefulness after learning” was held constant. Subjects in the “2^nd^ night wake” group learned at 22:30 h and retrieval testing was performed 33 hours later at 08:00 h. They slept at home during the first night after learning and stayed awake the second night prior to the retrieval session in the sleep laboratory. Subjects in the “2^nd^ night sleep” group slept both nights between learning and retrieval. All subjects were instructed to go to bed at home between 22:00 and 00:00 h, to get rise between 06:00 and 08:00 h, and to sleep at least 7 hours. They wore Actiwatches® (Cambridge Neurotechnology Ltd) and filled in questionnaires to control for bedtimes and estimate total sleep time.

#### Experiment III

By comparing a “1^st^ night wake” and a “1^st^ night sleep” group, with retrieval testing performed after subjects had slept normally in the second night, only the factor “sleep vs. wakefulness after learning” was manipulated, while all subjects were not sleep-deprived at retrieval. Subjects in both groups learned at 22:30 h and recognition testing occurred 44 hours later at 18:30 h in order to match the time of day for learning and retrieval. During the first night, subjects in the “1^st^ night sleep” group slept in the sleep laboratory with polysomnographic recording. Subjects in the “1^st^ night wake” group were kept awake in the laboratory (as described for Experiment I). They went home at 07:00 h and were asked to stay awake until 20:00 h the next evening. Adherence to this instruction was confirmed by actigraphy. During the second night, subjects in both groups slept at home (see above) and filled in sleep questionnaires for bedtimes and total sleep time.

#### Experiment IV

This experiment investigated the influence of the adenosinergic antagonist caffeine on false memory generation at retrieval in subjects who were acutely sleep deprived. Subjects learned at 22:30 h and recognition performance was tested at 07:30 h the next morning. Between learning and retrieval all subjects stayed awake in the laboratory as described for Experiment I. One hour before the start of the recognition test, subjects were either administered a capsule containing 200 mg caffeine (“night wake caffeine”) or placebo (“night wake placebo”) according to a randomized, double-blind design.

### Control variables: subjective ratings, sleep data, and salivary cortisol

Prior to learning and recognition testing, subjects in all experiments rated their subjective sleepiness, activation, motivation and concentration on 5-point Likert-scales with 1 indicating “not at all” and 5 indicating “very much”. In Experiment IV subjects additionally filled in a caffeine symptom questionnaire after recognition testing ([62]; 20 items, each ranging from 0 = “not at all” to 3 = “very much”). “Caffeine effect scores” differed significantly between the “night wake caffeine” and “night wake placebo” groups [14.27±3.07 vs. 5.39±1.35, t (31) = 2.80, P = 0.016]. Sleep quality in Experiments I to III was controlled by standard polysomnographic recordings and sleep questionnaires, as detailed above. Polysomnographic recordings were visually scored as wake or stages 1, 2, 3, 4 and REM sleep according to standard criteria [63].

In Experiment I, salivary cortisol concentrations were additionally measured immediately before, during and after recognition testing to control for possible influences of circadian and awakening-related variations in glucocorticoid release known to influence memory function [34], [64].

### Statistical analysis

Data from subjects whose false memory performance differed from the group mean by more than two standard deviations were identified as outliers and excluded from analysis. This criterion applied to four subjects whose false memory rate was drastically below the group mean [one subject was removed from each of the following groups: night wake (Exp. I), 2^nd^ night wake (Exp. II), 1^st^ night sleep (Exp. III), night wake caffeine (Exp. IV)]. Data from two subjects had to be rejected because one performed completely at chance level (∼50% hits, false memories and false alarms), and the other had almost 100% hits, false memories and false alarms, making an adequate evaluation of memory performance impossible.

Memory data were analysed by standard procedures of recognition memory analysis (see [25]). Basically, this analysis uses false memory rates, hit rates and false alarm rates as the primary dependent variables, which were corrected prior to analysis as recommended by Snodgrass and Corwin [25] to account for deviations from the normal distribution due to positive skewness. Additionally, to correct for baseline propensity to accept items the discrimination indices P*_r_* and bias indices B*_r_* were computed for hits and false memories according to the Two-High-Threshold model (i.e., correct recognition: P*_r_* = hit rate−false alarm rate, false recognition: P*_r_* = false memory rate−false alarm rate; B*_r_* = false alarm rate / (1−P*_r_*), for correct and false recognition, respectively; see [25]). Measures were compared between groups using one-way analyses of variance (ANOVA) in Experiment I, and pairwise t-tests for independent samples in Experiments II to IV. Confidence ratings and Remember/Know/Guess judgments were analysed using a 3 (group)×3 (word type) ANOVA in Experiment I, and 2 (group)×3 (word type) ANOVAs in the Experiments II to IV. “Group” served as between-subjects factor and “word type” (list words, distractors, theme words) as within-subjects factor. For analyses of subjective ratings a 3 (group)×2 (session) ANOVA in Experiment I, and 2 (group)×2 (session) ANOVAs in Experiments II to IV were conducted, with “group” as between-subjects factor and “session” (learning vs. retrieval) as within-subjects factor. In case of significant effects, post-hoc pair-wise t-tests were computed. When appropriate, Greenhouse-Geisser correction of degrees of freedom was applied. The significance level was set to P = 0.05, two-tailed (except for false memories in Experiment II which were tested one-tailed due to a directional hypothesis).
